# Eating and swallowing care disparities in persons with dementia: A conceptual framework

**DOI:** 10.1002/alz.70028

**Published:** 2025-02-26

**Authors:** Raele Donetha Loy, Nicole Rogus‐Pulia, Fred Ketchum, Michelle Troche, Anaïs Rameau, Harrison N. Jones, Luis Riquelme, Andrea Gilmore‐Bykovskyi, Manish N. Shah, Amy Kind

**Affiliations:** ^1^ Department of Medicine School of Medicine and Public Health University of Wisconsin–Madison Madison Wisconsin USA; ^2^ Center for Health Disparities Research University of Wisconsin–Madison Madison Wisconsin USA; ^3^ Geriatric Research Education and Clinical Center William S. Middleton Memorial Veterans Hospital Madison Wisconsin USA; ^4^ Department of Neurology School of Medicine and Public Health University of Wisconsin–Madison Madison Wisconsin USA; ^5^ Laboratory for the Study of Upper Airway Dysfunction Teachers College Columbia University New York New York USA; ^6^ Sean Parker Institute for the Voice Department of Otolaryngology ‐ Head and Neck Surgery Weill Cornell Medical College New York New York USA; ^7^ Department of Head and Neck Surgery & Communication Sciences Duke University School of Medicine Durham North Carolina USA; ^8^ Department of Orthopedics & Rehabilitation Maimonides Medical Center Brooklyn New York USA; ^9^ Department of Neurosciences & Learning Universidad Catolica del Uruguay Montevideo Uruguay; ^10^ Teachers College Columbia University New York New York USA; ^11^ BerbeeWalsh Department of Emergency Medicine School of Medicine and Public Health University of Wisconsin–Madison Madison Wisconsin USA

**Keywords:** Alzheimer's disease, dementia, dysphagia, health equity, social determinants of health

## Abstract

**INTRODUCTION:**

Eating and swallowing difficulties are prevalent and distressing among persons living with dementia (PLWD). These challenges may be especially burdensome for PLWD in lower‐resourced settings, where environmental factors such as social support, health‐care infrastructure, and food access are critical for meeting quality standards of eating and swallowing care. However, clinical practices and research methods have not sufficiently focused on the lived environment to promote high‐quality, socially and culturally aligned management approaches.

**METHODS:**

To address this gap, we developed a conceptual framework informed by the literature, grounded in ecological systems and fundamental cause theories, and refined through iterative discussion.

**RESULTS:**

Our framework highlights individual‐, system‐, and community‐level factors and resources influencing person‐centered eating and swallowing care for PLWD. It identifies areas at risk for inequitable care along the swallowing management continuum.

**DISCUSSION:**

We propose future research areas to help health‐care providers reconcile the demands of eating and swallowing care with the lived realities of PLWD.

**Highlights:**

- There are eating/swallowing care disparities among persons living with dementia.
- We introduce a conceptual framework applying social and structural determinants of health to eating/swallowing care.
- We also recommend areas to address disparities and improve eating/swallowing care.

## 1 BACKGROUND

Dysphagia, or difficulty swallowing, is common among persons living with dementia (PLWD), affecting ≈ 86% of those diagnosed.^1^ Dysphagia is associated with serious, adverse health outcomes, including hospital stays that are ≈ 40% longer,^2^, ^3^ a 3‐fold increase in the risk of aspiration pneumonia,^4^ a 33.2% higher likelihood of transfer to a post‐acute care facility,^5^ and a 13‐fold increased mortality risk during hospitalization.^2^ As a result, individuals with dysphagia and their care partners face increased economic and personal burdens, greater health‐care use, and a reduced quality of life.^5^, ^6^ Supporting swallowing and eating‐related care needs becomes increasingly skill and time intensive as dementia progresses, including providing feeding support during mealtimes and introducing techniques to optimize swallowing function. Further, as highlighted in a recent qualitative study, health‐care providers and care partners often face significant challenges in making eating and hydration decisions for PLWD as the disease progresses.^7^ These challenges arise from the need to navigate individual‐level factors, such as personal food preferences and cultural practices, and broader factors, including hospital policies, institutional values, and legal constraints.^7^ If these factors are not adequately considered or act as barriers during the decision‐making process, they can hinder the delivery of optimal swallowing and eating care for PLWD.

Dementia care occurs in the context of care delivery systems with unevenly distributed resources, the impacts of which are well documented.^8^, ^9^ These disparities may be particularly relevant to eating and swallowing care, as supporting an optimal eating experience involves a complex, three‐phase process that imposes significant burdens at the individual, systems, and community levels. In the first phase, screening, swallowing concerns are identified by health‐care providers via patient report, questionnaires, or clinical screening tools. The second phase, diagnosis, involves evaluation by speech–language pathologists (SLPs), who use clinical swallowing evaluations and/or instrumental methods to characterize swallowing function. The third phase, treatment, involves patients receiving recommendations to manage their swallowing issues within their care setting. Each phase is influenced by numerous multi‐level factors, such as health‐care access, transportation, social support, insurance coverage, and provider availability. PLWD and their care partners often shoulder the burden of navigating these barriers to achieve optimal care.

Initiating eating and swallowing care in the outpatient setting relies on having access to a primary care provider (PCP) or specialist who can screen for symptoms and make a referral to a SLP—assuming the PLWD also has access to SLP services. Although instrumental approaches (e.g., videofluoroscopic swallow study) are considered advantageous during the diagnostic phase as they allow for direct visualization of swallowing function,^10^ their accessibility depends on various factors, including the ability of the PLWD to attend and afford a radiology appointment and the availability of equipment and trained personnel. The treatment phase typically involves implementing standard‐guideline approaches such as environmental and dietary modifications. Dysphagia researchers and clinicians have given little attention to equitable dysphagia care in the clinical and research domains. Current guideline approaches operate as “one size fits all” models that typically overlook unique cultural, social, economic, and geographic needs associated with eating and swallowing care. PLWD who reside in resource‐poor environments or have social and/or cultural practices not reflected in existing guideline‐concordant swallowing treatments may struggle to adhere to long‐term swallowing recommendations.^11^ This may be especially true for food intake modifications, which serve as a cornerstone of dysphagia treatment for PLWD^12^ but currently lack person‐centered options. All PLWD deserve opportunities for high‐quality, standardized care. Therefore, new approaches are urgently needed to maintain high standards of eating and swallowing care while introducing socioculturally tailored strategies that meet the diverse needs of PLWD.

A first step toward more person‐centered eating and swallowing care for PLWD involves adopting a social exposome perspective. The social exposome quantifies lifetime exposures in the lived environment and their effects on health.^13^ Within this framework, social determinants of health (SDOH) are viewed as the fundamental “causes of causes”—upstream non‐medical health factors such as income, education, employment opportunities, and housing quality influencing health outcomes.^14^ Over time, the concept of SDOH has expanded to include structural determinants of health (S/SDOH), which represent broader socioeconomic and political practices contributing to the unequal distribution of SDOH.^15^ These concepts underscore that experiences within the social environment are inseparable from health, necessitating consideration to ensure that eating and swallowing care approaches meet the standard of care for all individuals. By applying S/SDOH to dysphagia research in PLWD, we can gain a comprehensive understanding of eating and swallowing care outcomes. This approach also opens opportunities to identify barriers that may pose challenges navigating the eating and swallowing care process, along with options for socially and culturally aligned care and intervention. Given that dysphagia is common in PLWD, the absence of such tailored approaches represents an important gap in the field.

**FIGURE 1:**
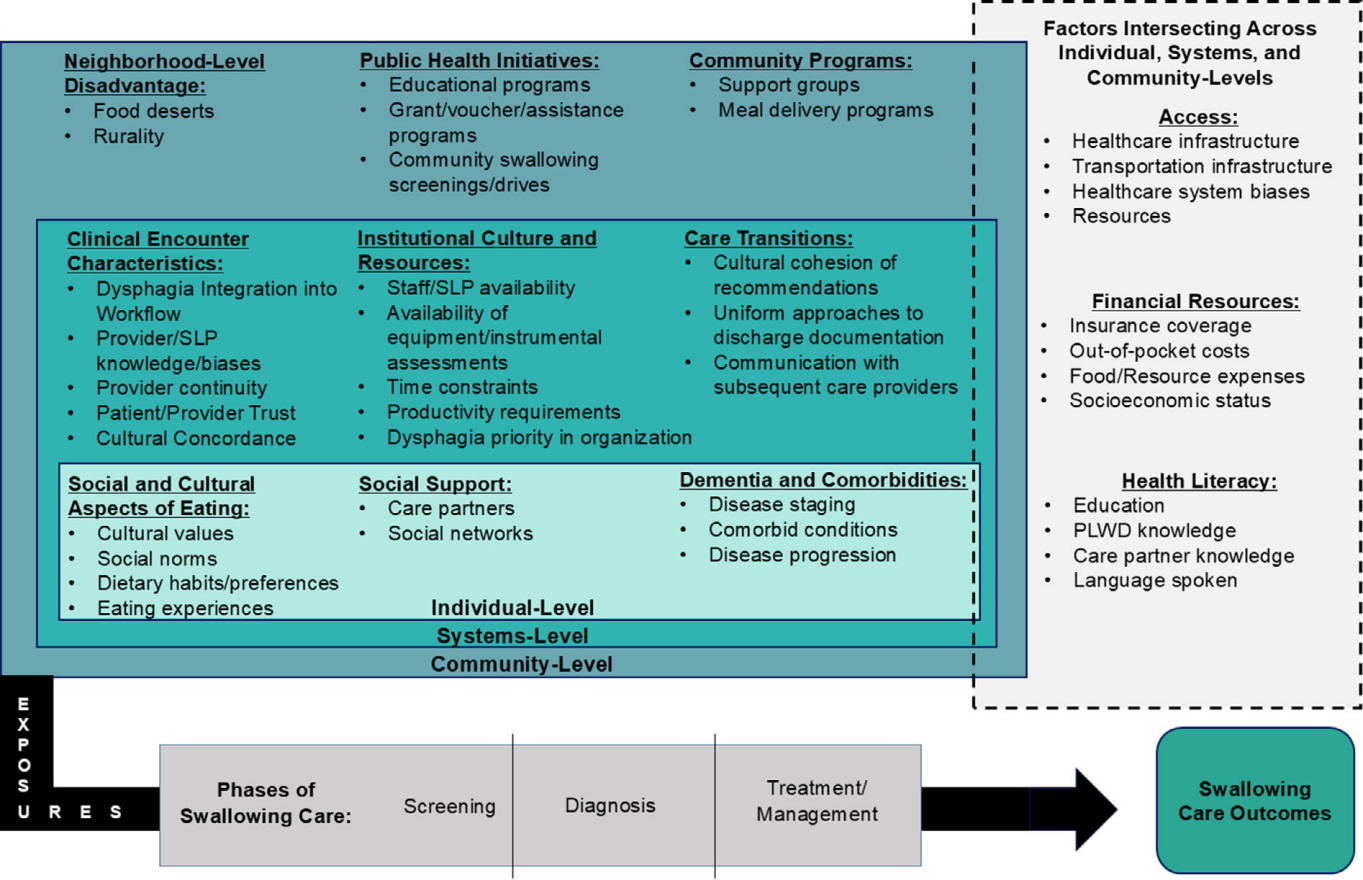
A novel conceptual framework of structural and social determinants of eating and swallowing care disparities in PLWD. PLWD, persons living with dementia; SLP, speech–language pathologist.

Our multidisciplinary team, in collaboration with several national experts in health disparities, dementia, and dysphagia, developed an initial conceptual framework that highlights multilevel health disparities impacting the phases of eating and swallowing care (Figure 1). We derived our framework from standard dysphagia care pathways for PLWD and the well‐established ecological systems^16^ and fundamental cause^17^ theories. Recent work by Adkins‐Jackson et al. applied the ecological systems theory to Alzheimer's disease (AD) and related dementias.^15^ Here, we apply the theoretical lenses of ecological systems^16^ and fundamental cause^17^ theories specifically to eating and swallowing care among PLWD.

In the following sections, we provide an overview of S/SDOH as it relates to dysphagia, outlining the individual, systems, and community‐level factors that influence the three general phases of eating and swallowing care. We also suggest potential future directions for equity‐focused eating and swallowing care research for PLWD.

## 2 METHODS

### 2.1 Overview of the structured process to develop the conceptual framework

The conceptual framework was developed through a structured, three‐step process. First, we grounded the framework in well‐established socioecological and health disparities models widely used in other fields. The ecological systems theory^16^ was chosen to highlight environmental influences on health outcomes while the fundamental cause theory^17^ allowed us to characterize specific factors and exposures within the environment that are relevant to swallowing and eating care. Next, from June 2023 to April 2024, the framework was refined through iterative discussions led by the first author (R.D.L.), who has expertise in swallowing and eating care for older adults with neurodegenerative disorders, and the senior author (A.K.), an expert in health disparities and the social exposome. Drawing on their expertise and the literature, they applied these models to existing clinical processes in swallowing and eating care. Finally, we convened a multidisciplinary group with expertise in speech–language pathology, otolaryngology, neurology, emergency medicine, dysphagia, dementia, and health disparities to review and provide feedback on the framework. Feedback from this group, collected during two phases (April–June 2024 and October–November 2024), informed revisions that led to refinement of the final version of the framework.

**RESEARCH IN CONTEXT**

1. **Systematic review**: Given the limited literature on health disparities in dysphagia (difficulty eating and swallowing), and the absence of articles explicitly addressing eating and swallowing care disparities in persons living with dementia (PLWD), we drew from broader dementia care disparities literature to inform our work.
2. **Interpretation**: We propose a conceptual framework that considers the role of structural and social determinants of health in eating and swallowing care disparities among resource‐poor PLWD. Our model uses the fundamental cause theory to characterize essential resource disparities that can hinder the provision of equitable eating and swallowing care. We also use the ecological systems theory to describe how these disparities emerge at the individual, systems, and community levels.
3. **Future directions**: We highlight three critical areas (research, resources and training, and socioculturally aligned dysphagia treatments) needing increased attention to develop pathways toward more person‐centered and equitable eating and swallowing care for PLWD.

### 2.2 Components of the conceptual framework

Our conceptual framework (Figure 1) contextualizes swallowing and eating care within the organizational structure of the social exposome as outlined in previous research.^13^, ^14^ The social exposome considers general (e.g., climate and traffic patterns) and specific exposures (e.g., dietary habits and physical activity) and their effects on health.^14^ It includes physical and social environmental exposures, such as cultural values, social interactions, and public policies.^13^, ^14^, ^18^ In our framework, we operationalize literature‐informed exposure pathways into social exposures to highlight their effect on eating and swallowing care outcomes. We accomplish this by using the ecological systems^16^ and fundamental cause^17^ theories.

The ecological systems theory^16^ posits that the environment consists of multiple layers, helping us understand the different levels involved throughout the phases of eating and swallowing care where disparities may occur. Our framework includes three levels: (1) “individual,” representing proximal factors relevant to PLWD, such as eating preferences and comorbid conditions; (2) “systems,” representing broader organizational and institutional structures, policies, values, and practices; and (3) “community,” representing widespread socioeconomic conditions, programs, and policies that affect the broader population. Importantly, the ecological systems theory emphasizes interactions that occur within and across levels. Highlighting these interactions is a key component of our framework, as it allows for recognition of how exposures at one level may influence outcomes at another level. For example, the community‐level factor of residing in a food desert may influence individual‐level dietary habits. To this end, our conceptual framework emphasizes the intersecting nature of individual‐, systems‐, and community‐level factors.

Link and Phelan's fundamental cause theory^17^ informs our understanding of exposures occurring within each level that may influence eating and swallowing care. This theory suggests that the persistent association between disproportionately impacted population groups and mortality arises from individuals having fewer flexible resources—such as money and power—to protect and improve their health.^17^ Within our framework, we identify broader system factors and resources such as organizational culture and staffing shortages that may affect the eating and swallowing care process at each surrounding level. We identify several factors, including access, financial resources, and health literacy, that are relevant resources across each level. These factors are depicted as a gray box overlapping each level in the framework (Figure 1).

## 3 RESULTS

### 3.1 Individual‐level exposures

#### 3.1.1 Social and cultural aspects of eating

Social and cultural factors significantly influence eating experiences throughout an individual's lifetime. People may develop specific dietary habits and preferences based on their religion, social norms, and the region in which they live. Social events, including holidays and weddings, often revolve around food traditions, and cultural norms can affect how food is consumed, such as with a fork or by hand. Considering these social and cultural expectations in eating and swallowing care is crucial, as they may impact PLWD's decisions to seek care and the types of treatments that may or may not be acceptable.^19^ PLWD may refuse treatments that misalign with their religious, social, and/or cultural practices. For instance, SLPs frequently recommend diet modifications, such as changing food consistency, for PLWD.^12^ However, foods like pureed meats and vegetables may be unfamiliar and inconsistent with specific cultural practices regarding how foods are prepared and consumed. While there are currently no accepted guidelines in the field of dysphagia on how to best approach these considerations when making dietary adjustments, a few prior works offer some recommendations.^20^, ^21^, ^22^, ^23^ As an initial step, health‐care providers are encouraged to adopt cultural humility practices, which involve self‐reflection and self‐critique of current approaches and interactions with patients with diverse needs, as well as actively seeking opportunities to learn about their unique sociocultural needs.^21^, ^23^ Using ethnographic interview techniques to better understand sociocultural factors such as food preferences and family dynamics may also help guide clinical decision making surrounding dietary adjustments.^21^, ^23^ In a case study examining the ethics of food and culture in dysphagia management, Kenny^24^ found that shared decision making can be an effective clinical approach to navigating diet decisions. Kenny suggests shared decision making is most successful when there is open communication between patients and providers; exploration of alternative strategies in cases in which recommendations are misaligned with sociocultural preferences and needs; advocacy for patients to express their needs;, and self‐reflection on personal values, biases, and assumptions that may negatively influence clinical decision making.^24^

#### 3.1.2 Social support

A recent survey study examining SLP dysphagia management in PLWD found that the most common dysphagia treatment used “always” or “most of the time” was care partner education.^12^ This finding underscores the critical role of social support in the eating and swallowing care of PLWD. Care partners often assume the primary responsibility for managing eating and swallowing care in the home environment. Their tasks may include purchasing food and beverages for meals, preparing meals (e.g., pureeing food), setting medical appointments, transporting PLWD to these appointments, and assisting PLWD with eating during mealtimes. These increasing responsibilities likely contribute to the heightened burden experienced by care partners of PLWD with dysphagia.^6^ Some PLWD have social support readily available and rely on spouses and children for assistance.^25^ Others without immediate family may turn to community members, such as friends, neighbors, and church parishioners.^26^ However, PLWD who lack social support altogether may find it challenging to meet their eating and swallowing needs.

#### 3.1.3 Dementia and comorbid conditions

The progression and staging of dementia, along with the presence of comorbid conditions, may influence the extent of individual, systems, and community resources needed for eating and swallowing care. Alterations in taste and smell among PLWD can affect food preferences with some PLWD noted to like new foods or dislike previously enjoyable foods as the disease progresses.^27^ Eating habits may change, requiring increased support from care partners and/or residential setting staff during mealtimes. This change could involve offering finger foods versus those requiring silverware^27^ or the PLWD receiving nutrition via a feeding tube.^1^ While enteral nutrition is generally no longer recommended for late‐stage PLWD due to increased risks with little nutritional benefits,^28^ recent qualitative work identified different social and cultural viewpoints influencing PLWD and care partner decisions around feeding tube placement.^29^

Historically, dysphagia management in PLWD has relied on reactive rather than proactive approaches,^30^ largely due to the longstanding view that dysphagia is a late‐stage consequence of dementia. However, prior work indicates that swallowing impairments can begin early in the disease course.^31^ Additionally, Kai et al. provided preliminary insights into how eating difficulties may evolve during disease progression.^32^ They found greater frequency of self‐reported swallowing and eating difficulties with advancing AD progression according to the Clinical Dementia Rating scale.^33^ Appetite changes were the most common issue in individuals with mild (49.5%) and severe (77.6%) AD, while changing eating habits (60.3%) were most frequently reported in those with moderate AD.^32^ Notably, “swallowing disturbances” were reported most often in individuals with severe AD (53.4%) compared to those in the mild (22.2%) or moderate (28.6%) stages.^32^ Misconceptions about the timing of dysphagia or lack of understanding of early swallowing and eating changes in PLWD may result in PLWD in the earlier stages receiving suboptimal eating and swallowing care, as symptoms are less apparent and, thus, screened or discussed less frequently. Consequently, eating and swallowing care and interventions are implemented reactively in the later stages of disease progression, when symptoms become more pronounced.

Still, providing eating and swallowing care to PLWD in advanced stages can also be challenging, as care complexities and resource demands increase with disease progression. PLWD at the more advanced stages of the disease often require palliative care approaches which may complicate decision making specific to swallowing and eating. In a recent paper discussing palliative care for patients with dementia, Malhi et al. describe how the negative impacts of swallowing and eating changes in PLWD may be mitigated by initiating palliative care early.^34^ Early integration of palliative care can support advanced care planning and goals‐of‐care discussions, allowing PLWD to express their needs and preferences while they are still capable of making decisions.^34^ Health‐care use and resource needs for PLWD with comorbidities may also increase due to the involvement of additional subspecialists, appointments, and therapies to address multisystem issues. In sum, PLWD in advanced dementia stages, or those with comorbid conditions and limited resources, face distinct disadvantages in addressing swallowing symptoms as they have fewer resources to allocate across their multifaceted care needs.

### 3.2 Systems‐level exposures

#### 3.2.1 Clinical encounter characteristics

Health‐care clinical encounters are central to eating and swallowing care. During interactions with PLWD and their care partners, medical providers—including primary care clinicians, geriatricians, nurses, nurse practitioners, neurologists, and other prescribing providers—play a critical role by conducting simple swallowing screens, initiating referrals to SLPs, and overseeing care plans and treatments. SLPs, in turn, use their clinical encounters to further screen, diagnose, and manage swallowing difficulties. Given these roles, it is imperative that screenings for eating and swallowing impairments and discussions around these issues are included in clinical encounters with these providers. However, our recent work (Loy et al., submitted) found the greatest barrier to dysphagia screening among health‐care providers in geriatric medicine was a lack of integration of dysphagia in the clinical workflow. Further compounding this, we also found many health‐care practitioners (74%) perceived their dysphagia‐related training and education to be insufficient. Similarly, SLP dysphagia training can vary, with dysphagia only being taught as an elective or a single‐semester course in some areas.^35^ These gaps in dysphagia knowledge and emphasis within the clinical encounter may be substantial, leaving some PLWD under the purview of providers lacking a sufficient ability to identify and manage swallowing concerns.

In addition to training disparities, maintaining consistency in dysphagia care presents a significant challenge across health‐care settings. In safety net systems, such as Federally Qualified Health Centers (FQHCs) and rural critical access hospitals, provider turnover and reliance on locum tenens or temporary providers^36^ can create variability in dysphagia expertise and care management. As eating and swallowing care is ongoing, PLWD who lack consistent interactions with the same health‐care provider may experience significant differences in how dysphagia is managed and how well their individualized care plan is upheld.

The clinical encounter between PLWD and health‐care providers may also be influenced by sociocultural characteristics. The field of SLP is predominantly White (90.5%),^37^ hindering the capacity to deliver socioculturally concordant dysphagia care for diverse PLWD. Culturally concordant health care can improve patient satisfaction, trust, and intent to adhere to treatments.^38^ However, historical injustices in health care and medical research have fostered mistrust in many minoritized groups,^39^ who may feel wary of their providers as a result. Limited diversity among SLPs may intensify this mistrust, creating additional barriers to effective eating and swallowing care. These clinical encounter characteristics illustrate how disparities in dysphagia training, integration within clinical workflows, care continuity, and SLP workforce diversity may contribute to inequitable eating and swallowing care for some PLWD.

#### 3.2.2 Institutional culture and resources

Eating and swallowing care may vary significantly across institutions due to differences in organizational culture and available resources. Our research identified working in institutions that prioritize dysphagia as a key facilitator to integrating swallowing screening into geriatrics clinics (Loy et al., submitted). Diagnosing dysphagia typically requires instrumental assessments; however, not all facilities have access to the equipment for these assessments or the necessary staff, such as radiology technicians. A study by Morton et al.^40^ revealed that in rural Alabama, most facilities (85%, *n* = 52) had only one to two medical SLPs, and in the county with the most SLPs (*n* = 11), all were employed by nursing homes. SLP availability is generally limited, with ≈ 180,000 certified SLPs employed nationally,^37^ compared to another rehabilitation specialty, physical therapy, which employs > 500,000 physical therapists and assistants nationwide.^41^ Among the national pool of SLPs, only 39.6% work in hospitals, 8% in residential health care, and 19.5% in non‐residential health‐care settings.^37^ Even when providers are available, they must have sufficient time for comprehensive eating and swallowing care, including discussions about eating, performing screenings, conducting detailed assessments, and developing personalized management plans. However, time constraints and lack of specialist support can hinder quality care for PLWD,^42^ and might be especially detrimental to those with dysphagia in need of these additional resources. PLWD receiving eating and swallowing care in settings with staff shortages, increased productivity requirements, time constraints, and where dysphagia is not prioritized are likely at a disadvantage.

#### 3.2.3 Care transitions

Our recent retrospective analysis found that, while 85% of inpatients with dementia from neighborhoods with increased socioeconomic disadvantage were given swallowing recommendations that may require extra resources, there was no documentation these patients were provided with SDOH counseling or resource linking before discharge.^11^ This finding reflects a failure to recognize and address unmet needs of PLWD in the home environment, potentially impacting their ability to continue eating and swallowing care after discharge. Our recent work, combined with prior findings from Kind et al. that found high rates of SLP recommendation omissions on discharge summaries,^43^ suggests there may be broader, system‐level inequities to supporting eating and swallowing care transitions among PLWD. Those receiving eating and swallowing care in health‐care systems with SLPs lacking knowledge/education about S/SDOH or without systematic approaches for identifying socially vulnerable patients will likely have their needs go unaddressed. Providers and health‐care systems with suboptimal means of communicating pertinent swallowing information and recommendations via standardized approaches for discharge summary documentation or by engaging providers at subsequent care facilities will also place PLWD at a disadvantage for experiencing continuity of eating and swallowing care. Furthermore, critical information related to swallowing and eating care, such as preexisting difficulties and mealtime adaptations implemented in the home environment, may not be communicated to health‐care team members during care transitions. Such lack of communication can lead to a reduced ability of the health‐care team to adequately respond to swallowing and eating care needs across settings. These points underscore the importance of a comprehensive approach to care transitions, which must involve coordinated efforts between multiple health‐care providers—including social workers, doctors, and SLPs—as well as health‐care systems equipped with the resources, standardized procedures, and infrastructure necessary to address social vulnerabilities and support effective community reintegration for PLWD.

### 3.3 Community‐level exposures

#### 3.3.1 Neighborhood‐level disadvantage

The neighborhood and built environment are proximal to the individual during the life course and offer infrastructure and resources such as health‐care settings, green spaces, parks, pharmacies, and grocery stores that directly impact health.^44^ As such, whether the structural and social features of the neighborhood constrain or enhance health‐related behaviors can directly affect health outcomes.^44^ Metrics like the Area Deprivation Index (ADI) characterize the social environment of a precise region to quantify neighborhood‐level socioeconomic disadvantage.^45^ In PLWD, these metrics have specifically demonstrated that individuals residing in neighborhoods with the greatest socioeconomic disadvantage have an increased risk of AD neuropathology^46^ and re‐hospitalization within 30 days^47^ as well as demonstrate poorer cognitive performance.^48^ These exposures to socioeconomic disparities may have implications for eating and swallowing care. For instance, PLWD living in a food desert or rural areas may have increased difficulty accessing resources necessary for their eating and swallowing care such as specialized food items and health‐care appointments and are more likely to face readmission.

#### 3.3.2 Public health initiatives

The benefit of applying a social exposome perspective to eating and swallowing care is that it allows us to understand swallowing‐related outcomes in terms of inequities within the social environment. This reframing allows us to approach eating and swallowing care as a public health concern rather than as a personal issue, emphasizing the need for policy changes that address S/SDOH. Historically, health care has not viewed eating and swallowing care from this perspective, leading to gaps in public health initiatives that provide education, resources, and support for those affected by dysphagia. Consequently, PLWD may rely on their family, friends/peers, or health‐care providers for information, potentially creating disparities for those whose networks lacks this knowledge. Furthermore, public health initiatives such as grants for thickener costs, free or low‐cost community swallowing screenings, and food assistance programs with dysphagia‐friendly foods are essential yet underdeveloped. Until policies and programs are developed and implemented to address these gaps, some PLWD, particularly those who are lower resourced, will continue to experience suboptimal and inequitable eating and swallowing care.

#### 3.3.3 Community programs

A recent mixed‐methods study examining dysphagia among hospitalized older adults with dementia found the majority of care partners wanted additional information about dysphagia management before discharge.^49^ One care partner in this study expressed that a community member provided additional knowledge regarding dysphagia, “otherwise, [they] would have been lost.”^49^ This finding suggests community social support may be an important tenet of ongoing eating and swallowing care for PLWD and their care partners when they leave the clinical setting. Similar to the survey participant, some PLWD and their care partners may seek support informally through their social network. Less is known about sources of formal dysphagia support available for PLWD, but may be found in support groups offered by organizations such as the Alzheimer's Association or through church groups, local events, and community programs. PLWD may also use meal delivery programs in their communities such as Meals on Wheels to fulfill their dietary intake; however, it is unclear how well these types of services currently meet the needs of those with dysphagia.^50^ PLWD in communities lacking programs where they can derive support and resources related to their eating and swallowing care needs may experience an increased burden compared to those living in enriched environments.

### 3.4 Resources across levels: access, finances, and health literacy

#### 3.4.1 Access

The three phases of eating and swallowing care rely on individual‐, systems‐, and community‐level access. PLWD who live alone or lack social support may have difficulty leaving home to gain essential items from the grocery store or attend health‐care appointments. PLWD with lower education or reduced health literacy may have difficulty accessing and understanding pertinent information about swallowing changes and care, potentially influencing their decision to obtain eating and swallowing care or reducing their ability to sufficiently address care needs. PLWD lacking access to health‐care systems or institutional resources have less opportunity to have their symptoms screened, diagnosed, and treated. From a broader community/public standpoint, PLWD residing in communities with increased socioeconomic disadvantage likely have fewer resources such as grocery stores and support programs, to manage their eating and swallowing care needs.

#### 3.4.2 Financial resources

Dysphagia management depends on many financial resources including personal income and medical insurance. Individual income is needed to cover the costs of food, copays, and out‐of‐pocket expenses for certain dysphagia treatments, such as blenders to puree food. Insurance coverage helps offset the costs of instrumental swallowing assessments (e.g., videofluoroscopy) to obtain a diagnosis. Insurance also helps cover health‐care costs including medical appointments, and inpatient and outpatient rehab. While most insurance plans will cover some or most of these costs, PLWD who are under‐ or uninsured may be unable to receive adequate eating and swallowing care. Some PLWD may use public programs such as Supplemental Nutrition Assistance Program to pay for food. The extent to which these types of programs cover the dietary needs of PLWD with dysphagia, for example by covering the costs of items like thickener, has not been sufficiently examined.

#### 3.4.3 Health literacy

Health literacy was found in a recent systematic review^51^ to encompass three important constructs: (1) knowledge of health, (2) the ability to process information related to health in various formats, and (3) the management of health through individual actions and by working with health‐care providers. These constructs of health literacy are essential to the eating and swallowing care process, with initial awareness of swallowing changes dependent on PLWD having access to/been provided with this information. Without PLWD and care partners having education materials as they progress through the eating and swallowing care process, they are at risk of not understanding or fully benefiting from procedures and diagnostics. Swallowing treatment and follow‐up care will be largely managed by PLWD and their care partners in the home environment, necessitating they are properly equipped with education and resources to successfully address their eating and swallowing‐related care needs when they leave the clinical setting.

## 4 DISCUSSION

The failure to consider S/SDOH in eating and swallowing care of PLWD contributes to persistent health inequities, placing socially vulnerable PLWD at a disadvantage to adequately address their eating and swallowing care needs. While this work is a starting point and there will be many future avenues of exploration, for now, there are three critical areas that need addressing: (1) research studies to examine unmet needs existing within the individual, systems, and community levels; (2) improved resources and training at the systems and community levels to better support person‐centered eating and swallowing care; and (3) improved eating and swallowing treatments that enhance options for person‐centered care practices by integrating S/SDOH.

### 4.1 Research

More disparities‐focused research is crucial for understanding dysphagia in PLWD. Epidemiological studies can shed light on the extent of under‐detection of dysphagia, examining disparities that affect PLWD's access to screening and diagnosis. These studies can also reveal who participates in each stage of eating and swallowing care and whether S/SDOH contribute to eating and swallowing care delays or attrition. Qualitative research is needed to engage PLWD and their care partners directly, providing insights into their experiences with eating and swallowing care throughout disease progression.

As health disparities rarely occur in isolation, research must integrate analyses across individual, systemic, and community levels to understand their compounded impact on eating and swallowing care. This approach will help us systematically assess barriers and facilitators to navigating eating and swallowing care. This foundational research will pave the way for developing socially and culturally tailored, person‐centered interventions and practices to achieve equitable standards of care for all PLWD. By generating this critical knowledge, we can advocate for comprehensive system and policy changes necessary to address eating and swallowing care inequities effectively. We recognize that similar research efforts are likely needed for other patient populations affected by dysphagia. We encourage future work to adapt the constructs in the current framework to guide research aimed at examining and addressing eating and swallowing care disparities in these populations.

### 4.2 Resources and training

Heightening expectations for socially responsive eating and swallowing care is only feasible if we implement increased resources and training at the system and community levels. These efforts will enable relevant stakeholders to effectively deliver and receive eating and swallowing care. Practitioners in various fields—including SLP, medicine, nursing, and pharmacy—can benefit significantly from enhanced dysphagia training during their education. Health‐care providers trained and encouraged to account for S/SDOH within their clinical practice may engage in more person‐centered care, by tailoring their approaches to their patients’ health literacy, culture, and living conditions. Educational, accessible initiatives specifically targeting health‐care providers involved in dysphagia management should be developed to enhance their capacity to deliver equitable swallowing services to PLWD.

Integrating health‐care systems with social resources will likely best facilitate person‐centered eating and swallowing care. Health‐care systems can use social resources including electronic health record templates embedded with relevant S/SDOH items, clinical toolkits that guide social and culturally responsive practices into workflows, and centralized and multidisciplinary resource hubs to help providers efficiently connect patients with the resources they need for eating and swallowing care. Broader health‐care policy changes can elevate the standards of clinical practice for PLWD with dysphagia. For instance, Medicare requires specific documentation elements, including objective measurements of patient performance and a certified plan of care, for reimbursement of SLP services.^52^ Setting minimum documentation standards that incorporate S/SDOH characteristics when developing dysphagia care plans can enhance accountability for identifying and addressing patient social needs. However, we acknowledge that these additional responsibilities may be challenging under certain health‐care models, such as fee‐for‐service systems, in which providers face increasing burnout due to time constraints and productivity demands. Therefore, comprehensive systems and policy changes are essential to equip health‐care providers with the necessary time and resources to incorporate S/SDOH‐aligned dysphagia practices into their clinical routines.

Supporting the transition of PLWD with dysphagia and their care partners across settings will require the development of additional systems‐ and community‐level resources. Interdisciplinary training models for care partners focused on food preparation and texture modification can help alleviate care partner burden. Further, patient care navigator programs, successfully implemented in fields like oncology,^53^ can be specifically tailored for PLWD with dysphagia to guide patients through the complexities of eating and swallowing care and address individual‐, system‐, and community‐level barriers. Establishing such programs may be particularly beneficial for socioeconomically disadvantaged PLWD who often lack access to essential resources.

### 4.3 Treatment

Enhancing the treatment of PLWD with dysphagia requires applying a social exposome lens to eating and swallowing care. First, health‐care providers and systems must acknowledge that health disparities operate at multiple levels throughout eating and swallowing care. Recognizing these disparities enables more personalized care with services tailored to the unique social, cultural, and economic conditions of PLWD and their care partners. Addressing disparities explicitly, rather than assuming a “one size fits all” approach, can lead to better health outcomes by ensuring that the specific needs of each individual are met.

Second, there is a critical need to develop culturally and socially responsible swallowing treatments. The treatment and follow‐up phases of eating and swallowing care are likely the longest, as eating and drinking are daily activities that require continuous management. Given PLWD and their care partners will primarily oversee these needs at home or in long‐term care settings, treatments must be socially and culturally aligned with their beliefs and values. Beyond cultural sensitivity, swallowing treatments must also be practical and feasible within the patient's environment to ensure long‐term adherence and successful outcomes. These types of tailored treatments will create a more effective and meaningful care process for PLWD.

These recommendations, while not exhaustive, aim to initiate further dialogue and research into the equity of swallowing treatments for PLWD—an area that has been overlooked for too long. Our conceptual framework indicates that barriers to culturally concordant eating and swallowing care exist across multiple levels, from individual providers to broader systems. Therefore, interventions that engage stakeholders across these levels are most likely to yield favorable outcomes for PLWD with diverse cultural and social needs.

## 5 CONCLUSION

Our conceptual framework offers a novel approach to understanding how health disparities arise and persist throughout the eating and swallowing care process for PLWD. Drawing from the social exposome concept, which examines S/SDOH surrounding an individual and their impact on health outcomes, our framework similarly explores S/SDOH across multiple levels to assess their effects on eating and swallowing care outcomes. By viewing eating and swallowing as a person‐centered activity influenced by S/SDOH, we can identify barriers and facilitators at various levels, gaining insight into how PLWD navigate the eating and swallowing care process and how disparities in their lived experiences can influence their care outcomes. Moreover, it is essential to understand the role of resources, institutional policies, and broader socioeconomic and political practices that shape the environments involved in eating and swallowing care. By doing so, we can uncover modifiable factors at these levels, creating opportunities for targeted interventions and informing broader policy changes to address eating and swallowing care disparities.

## CONFLICT OF INTEREST STATEMENT

Dr. Rogus‐Pulia is a VA employee and this work was partially supported by the William S. Middleton Veteran Affairs Hospital, Madison, WI, USA (X placeholders Manuscript 05‐2025). The content and views expressed in this article are those of the authors and do not necessarily reflect the position or official policies of the United States Government or the US Department of Veterans Affairs. Dr. Troche receives royalties from MedBridge, Inc. Dr. Rameau owns equity in Perceptron Health Inc. She is also a medical advisor for Sound Health Inc. Author disclosures are available in the supporting information.

## ROLE OF THE FUNDING SOURCES

The funding organizations were not involved in the design or conduct of the study; collection, management, analysis, or interpretation of the data; preparation, review, or approval of the manuscript; or decision to submit the manuscript for publication. The content is solely the responsibility of the authors and does not necessarily represent the official views of the National Institutes of Health.

## CONSENT STATEMENT

No human subjects were used in this work; thus, informed consent was not applicable.

## Supporting information



Supporting Information
